# Reducing time from presentation to surgical intervention for testicular torsion: implementation of a quality improvement protocol

**DOI:** 10.3389/fruro.2024.1383108

**Published:** 2024-07-16

**Authors:** Shannon Richardson, Kathy Huen, Tabitha Benga, Bridgette Fajardo, Renea Sturm, Steven E. Lerman, Jennifer S. Singer

**Affiliations:** ^1^ David Geffen School of Medicine at University of California, Los Angeles (UCLA), Los Angeles, CA, United States; ^2^ Department of Urology, David Geffen School of Medicine at University of California, Los Angeles (UCLA), Los Angeles, CA, United States

**Keywords:** testicular torsion, quality improvement, testicular salvage, pediatric urology, testis, orchiectomy, acute scrotum

## Abstract

**Introduction:**

Timely surgical intervention for patients with testicular torsion is a quality benchmark set by the U.S. News and World Report (USNWR) for pediatric urology. In this study, we describe and evaluate a quality improvement initiative to reduce the time to surgical intervention for testicular torsion at a single institution through the implementation of a clinical care pathway called “code torsion.”

**Methods:**

Data abstraction was performed through retrospective chart review to assess process measures. Patients <21 years old with testicular torsion requiring surgical intervention were included. The clinical protocol “code torsion” was created by a multidisciplinary quality improvement workgroup with the primary goal of reducing the time from emergency department presentation to surgical intervention for testicular torsion. “Code torsion” was implemented in October 2021, which was assessed in addition to subsequent interventions through plan–do–study–act (PDSA) cycles.

**Results:**

A total of 30 patients were identified prior to “code torsion” implementation and 14 thereafter. The mean time from triage to operating room (OR) was 228 min prior to “code torsion” compared with 180 min after protocol implementation (*p* = 0.047). The proportion of cases that had surgical intervention within the 4-h USNWR metric increased from 63% pre-protocol to 93% post-protocol (*p* = 0.07). Of the patients, 40% required orchiectomy prior to “code torsion” compared with 29% after implementation (*p* = 0.5). Patients requiring orchiectomy had a significantly longer time from symptom onset to surgical intervention (87 *vs*. 9.8 h, *p* < 0.001).

**Conclusion:**

Implementation of the protocol “code torsion” was successful in reducing the time from presentation to surgical intervention for testicular torsion. The rates of testicular salvage did not differ after “code torsion” implementation and were instead found to be dependent on the total ischemia time.

## 1 Introduction

Testicular torsion is a common urologic emergency with an annual incidence of 3.8 per 100,000 boys (1). If untreated, irreversible ischemia can develop, which could lead to decreased fertility or necessitate orchiectomy. The rates of testicular salvage are dependent on the time from symptom onset to detorsion (2, 3). As such, the U.S. News and World Report (USNWR) has recognized the time to surgical intervention within 4 h of presentation as a national pediatric urology quality metric.

The timely intervention for testicular torsion relies on various factors including the diagnosis, workup, and interdepartmental coordination. Thus, the time to triage, the diagnosis, and the communication between the emergency medicine, urology, ultrasound technicians, radiology, anesthesiology, and operating room (OR) staff all contribute to potential surgical delays. Prior studies have cited improvements in the emergency department (ED) presentation to the surgical detorsion time, also termed “door-to-incision time,” with interdepartmental interventions (4, 5). However, none have demonstrated improvement in the testicular salvage rates following quality improvement (QI) implementation (4, 5).

The aim of this QI initiative was to reduce the time to surgical intervention for testicular torsion at our institution through the implementation of a clinical care pathway developed through an iterative, multidisciplinary approach, called “code torsion.” Our primary outcome was door-to-incision time, with secondary endpoints including the proportion of cases that met the USNWR criteria of door-to-incision time of under 4 h, the testicular salvage rates, and the time differences in the presentation prior to and during the coronavirus disease 2019 (COVID-19) pandemic. We hypothesized that “code torsion” would improve the proportion of cases that met the USNWR criteria of door-to-incision time of under 4 h and increase the testicular salvage rates, and that patients would have delayed presentation during the COVID-19 pandemic.

## 2 Materials and methods

A multidisciplinary team was created as a QI initiative at our institution in response to the 2020 USNWR survey for pediatric urology. The QI team comprised pediatric urologists, emergency medicine physicians, OR staff, anesthetic, nursing, ultrasound (US) technicians, and clinical QI specialists. The primary goal was to decrease the door-to-incision time for testicular torsion, defined as the time from ED presentation to the time of surgical incision, within the 4-h USNWR metric at two hospitals within the same academic institution. A secondary aim was to assess the delays in symptom onset to ED presentation and the testicular salvage rates prior to and during the COVID-19 pandemic.

### 2.1 Data abstraction

We retrospectively identified male patients <21 years of age diagnosed with acute testicular torsion between 2019 and 2023 using previously published International Classification of Disease, Tenth Revision (ICD-10) codes (5). Only cases with enough clinical suspicion to warrant surgical scrotal exploration were included. Patients with neonatal torsion, intermittent torsion, return of vascular flow not necessitating emergency surgery (torsion–detorsion), or those who were not found to have torsion upon surgical exploration were excluded.

Process measures included the door-to-incision time, the time from triage to urology consultation, the time from scrotal US order to result, and the time from urology consultation to incision. Ischemia time was defined as the time from symptom onset to surgical intervention. Outcome measures included the proportion of cases that achieved surgical intervention within the 4-h USNWR metric and the rate of testicular salvage. While the signs of testicular salvage varied by surgeon from evidence of adequate Doppler flow to the subjective appearance of the testicle, this study defines testicular salvage as the absence of orchiectomy.

### 2.2 Primary drivers

**Figure 1:**
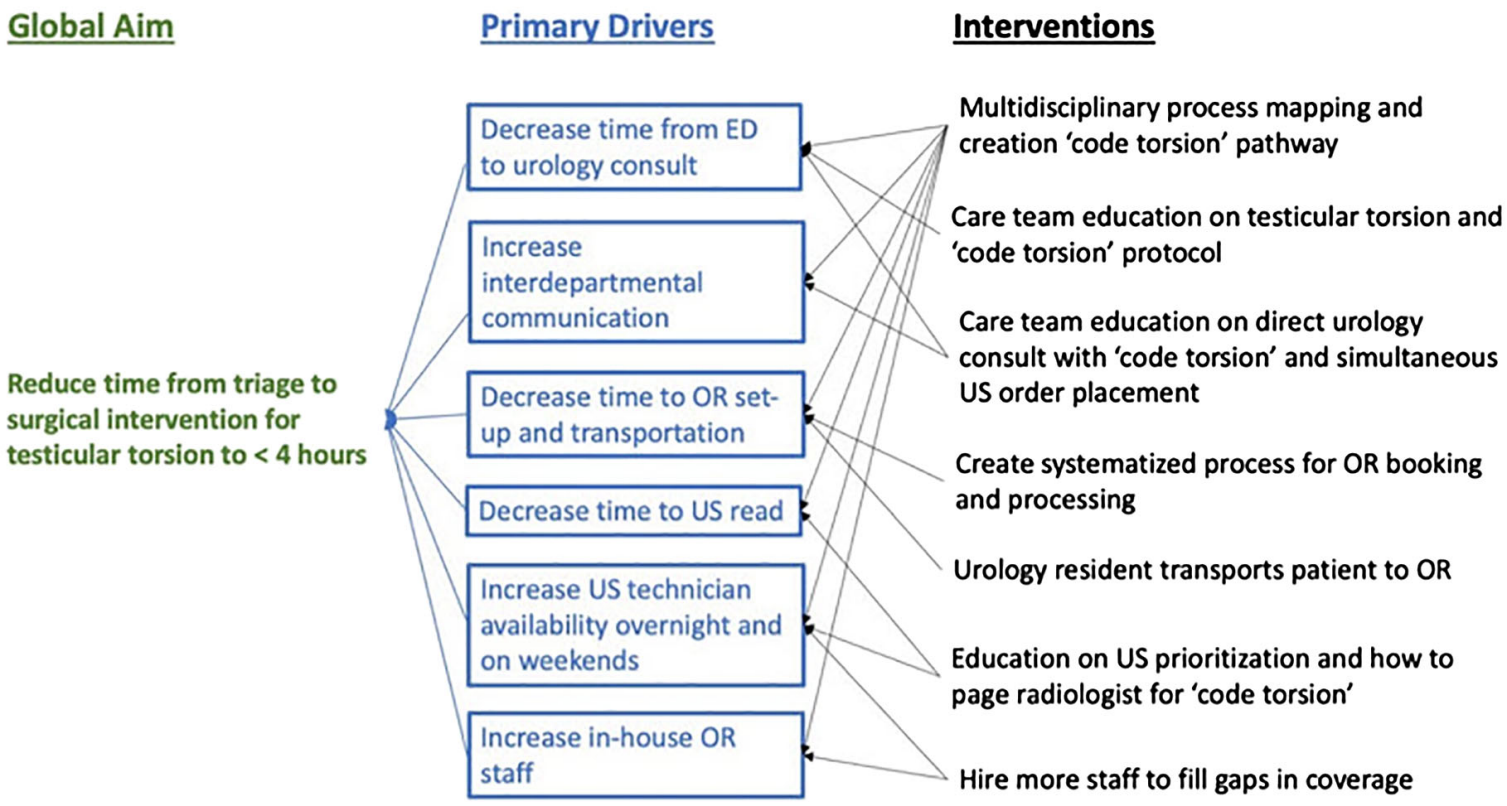
Global aim, primary drivers identified using a Pareto chart, and the interventions developed using plan–do–study–act cycles. ED, emergency department; OR, operating room; US, ultrasound.

After the creation of the interdisciplinary workgroup in August 2021, the primary drivers were identified using a Pareto chart (6). The three major contributors to the delay in the door-to-incision time were: 1) the time from ED triage to urology consultation; 2) the time from scrotal US check-in to result; and 3) the time from urology consultation to OR. A driver diagram was developed collaboratively within the workgroup (**Figure 1**). Specifically, at one of the two hospitals, the time from US to OR on weekends and at nighttime were identified as an additional delay. Since the urology resident would often read the US with the technician and book the case prior to the final read, the time to the radiology report did not necessarily contribute to delays in the door-to-incision time.

### 2.3 Implementation

**Figure 2:**
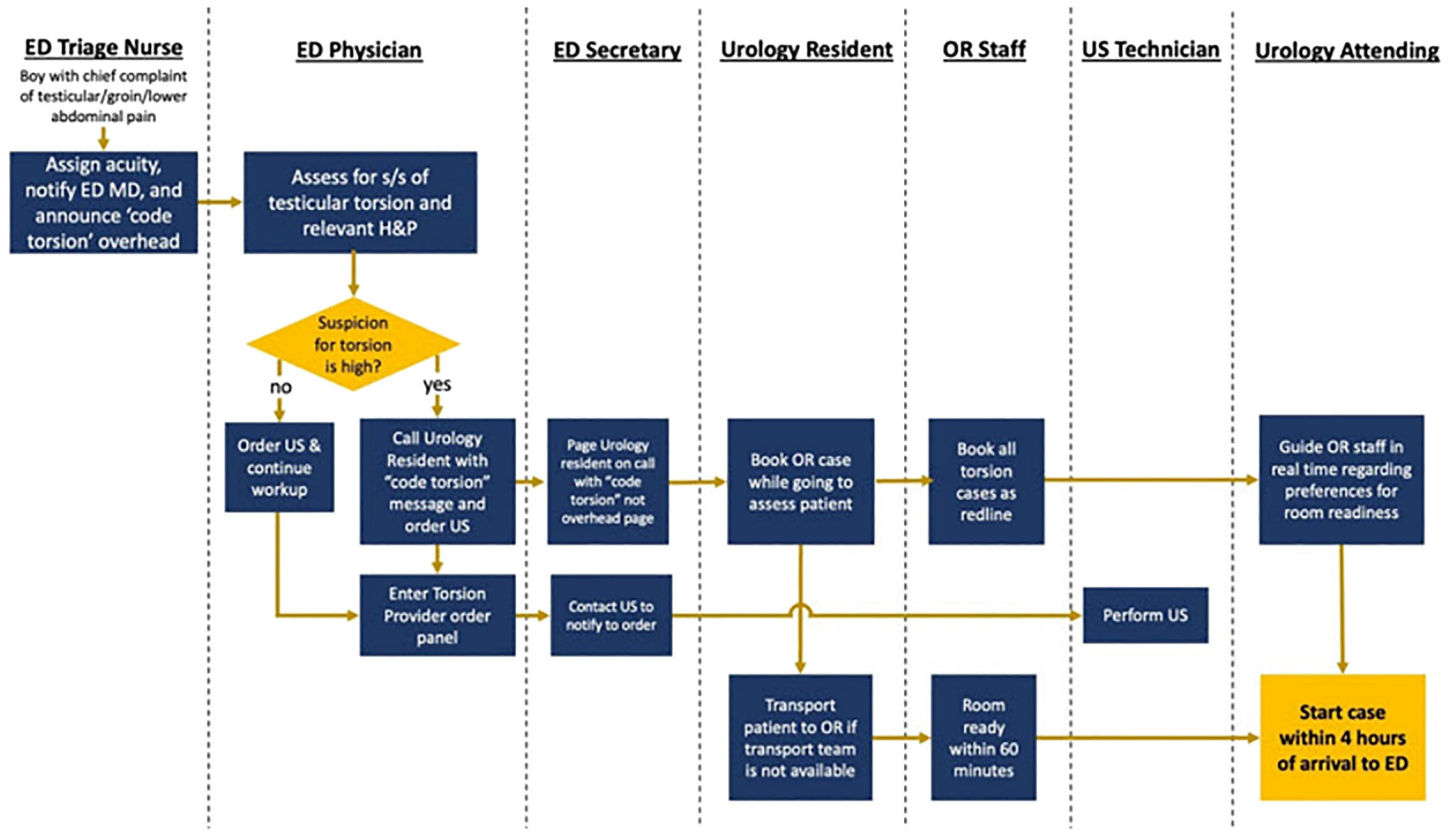
“Code torsion” protocol created by a multidisciplinary quality improvement team based on primary drivers and through process mapping. ED, emergency department; OR, operating room; S/S, signs and symptoms; US, ultrasound.

Plan–do–study–act (PDSA) cycles were utilized for real-time assessment and implementation (7). Interventions aimed at addressing these primary drivers were developed and synthesized into the “code torsion” clinical management pathway (**Figure 2**). The initial interventions included improving the ED triage nurse assignment of acuity and notification of the emergency medicine physicians (ED MD), the ED MD evaluation of the patient and simultaneously the consulting urology resident via the new ED classification, and overhead announcement of “code torsion” and ordering scrotal Doppler US, creating a systemic process for booking all cases as emergent/redline, meaning within 1 h from case booking, and including urology resident delivery of the patient to the OR when transport services are unavailable. Although the workgroup considered the use of the validated TWIST score, concerns regarding poor negative predictive values led to the ultimate consensus against using this decision support tool (5, 8). The “code torsion” pathway as shown in **Figure 2** was first implemented in October 2021.

Data analysis is ongoing with periodic workgroup meetings to determine the challenges and additional opportunities for intervention. For example, gaps in overnight and weekend US technician coverage persisted as a barrier after the initial implementation. Consequently, financial support was provided to hire additional US technicians.

### 2.4 Statistical analysis

Continuous variables were reported as medians with interquartile range (IQR) or means with standard deviations (SD), as appropriate, while categorical variables were presented as numbers with percentages. Wilcoxon’s rank sum and Fisher’s exact tests were used for bivariate comparisons. This study was deemed exempt from the University of California, Los Angeles, Institutional Review Board (IRB no. 23–001893) as it was categorized as a QI initiative. All statistical analyses were conducted using Stata version 16.1 software (StataCorp, College Station, TX, USA), with *α* set at 0.05.

## 3 Results

From January 2013 through October 2023, a total of 46 patients underwent scrotal exploration for suspected testicular torsion. On exploration, two patients did not have testicular torsion and were excluded. Of the remaining 44 patients, 30 were diagnosed with testicular torsion prior to implementation of “code torsion,” whereas 14 patients with torsion were identified after implementation. The patients’ characteristics are presented in **Table 1**. The median age of the pre-implementation group was 15 years (IQR = 14–17 years) compared with 14 years (IQR = 13–16 years) in the post-implementation cohort (*p* = 0.4).

**Table 1:** Patient characteristics stratified into pre- and post-implementation of “code torsion.”

	Pre-implementation (n=30)	Post-implementation (n=14)	*P*-value
**Median age, years [IQR]**	15 [14-17]	14 [13-16]	0.4
**Race, n (%)**			0.5
** Asian**	5 (17%)	0 (0%)	
** Black**	5 (17%)	4 (29%)	
** White**	10 (33%)	5 (36%)	
** Other/Missing**	10 (33%)	5 (36%)	
**Hispanic or LatinX, n (%)**	20 (67%)	3 (2%)	1.0

IQR, interquartile range.

The mean door-to-incision time was 228 min (SD = 78, range = 71–465 min) prior to “code torsion” compared with 180 min (SD = 57, range = 97–259) after protocol implementation (*p* = 0.047) (**Table 2**). The proportion of cases that met the USNWR metric of surgical intervention within 4 h increased from 63% pre-protocol to 93% post-protocol (*p* = 0.07) (**Table 2**).

**Table 2:** Outcome measures before and after implementation of “code torsion.”

	Pre- implementation (n=30)	Post- implementation (n=14)	*P*-value
**Mean door-to-incision time, mins (SD)**	228 (78)	180 (57)	0.047
**Door-to-incision time within USNWR metric** **< 4 h, n (%)**	19 (63%)	13 (93%)	0.07
**Mean time from ED arrival to urology consult, mins (SD)**	78 (54)	50 (51)	0.12
**Mean time from ED arrival to US order, mins (SD)**	26 (3)	19 (3)	0.3
**Mean time form ED arrival to US completion, mins (SD)**	108 (43)	91 (41)	0.3
**Ischemia time, h (SD)**	43 (67)	30 (40)	0.5
**Testicular salvage, n (%)**	12 (40%)	4 (29%)	0.5

USNWR, U.S. News and World Report.

The mean time from ED arrival to urology consultation decreased from 78 min prior to the QI initiative to 50 min post-implementation (*p* = 0.12) (**Table 2**). One common reason for the delays identified after the initial implementation was the prolonged time to urology consultation, which was addressed through improved education in the ED. The only case that did not meet the 4-h USNWR metric had a delayed time from ED triage to urology consultation of 2 h and 23 min.

Among the 44 patients, 37 (84%) had US performed prior to surgical exploration: 27 (90%) pre-implementation and 10 (71%) post-implementation. After the “code torsion” pathway, the mean time from presentation to US order decreased from 26 to 19 min (*p* = 0.3) (**Table 2**). Similarly, the mean time from ED arrival to US completion decreased from 108 to 91 min (*p* = 0.3) (**Table 2**).

There were 12 patients who required orchiectomy (40%) prior to “code torsion” implementation compared with 4 (29%) after implementation (*p* = 0.5). There was no significant difference in the ischemia time between the pre- and post-implementation groups (43 *vs*. 30 h, *p* = 0.5) (**Table 2**). Patients with testicular salvage had a significantly shorter mean ischemia time at 9.8 h (95% CI = 5–14) compared with those requiring orchiectomy (87 h, 95% CI = 47–127, *p* < 0.001) (**Table 2**). There were no differences in the ischemia time or the testicular salvage rates by race. Only 26% of the patients had a postoperative US performed during follow-up.

A total of 22 patients with testicular torsion were included prior to January 1, 2020, and eight patients presented during the COVID-19 pandemic. There was no difference in the mean door-to-incision time between the pre-COVID and COVID cohorts (228 *vs*. 227 min, *p* = 0.9). Patients with torsion during COVID delayed their presentation to the ED after symptom onset, with a mean ischemia time of 83 h compared with 28 h in the pre-COVID cohort (*p* = 0.05). Similarly, the testicular salvage rates were 38% during the COVID-19 pandemic and 68% prior (*p* = 0.13).

## 4 Discussion

Pediatric testicular torsion is a common urologic emergency. The time from the onset of symptoms to the surgical intervention for patients with torsion is critical for testicular salvage. The present study describes and assesses a QI initiative to reduce the time to surgical intervention for testicular torsion at a single institution. Utilizing a well-established root cause analysis methodology and a multidisciplinary team involvement, we successfully identified the key drivers causing delays in the door-to-incision-time and implemented a clinical care pathway called “code torsion” via PDSA cycles. Implementation of the protocol significantly reduced the time from presentation to surgical intervention and, although not statistically significant, increased the proportion of cases meeting the USNWR metric for testicular torsion. The rates of testicular salvage did not differ after “code torsion” implementation and were instead found to be dependent on the total ischemia time, which is similar to findings from previous studies (4, 5).

Prior studies have similarly created QI initiatives to target the time to surgical intervention for testicular torsion (4, 5, 9, 10). A single-center study by Zee et al. with a similar process mapping-based QI initiative reduced the median time to OR from 196 to 127 min (4). Another QI intervention that applied the TWIST score, a decision support tool for testicular torsion, found similar success in reducing the time from patient arrival to surgical intervention (5). These studies found no improvement in the testicular salvage rates or the ischemia time after QI implementation. We similarly found no difference in the ischemia time between the pre- and post-implementation cohorts.

A recent study assessing factors that impact the timing of testicular torsion management found delays in the presentation to the hospital as the most important contributor to ischemia time (11). Previous literature has also shown that various patient factors, such as age, lack of private insurance, and socioeconomic status, could contribute to pre-hospital delays (12–14). Taken together, our findings suggest that, while in-hospital delays can be optimized through clinical pathways such as “code torsion,” these interventions might not impact the ischemia time or the testicular salvage rates. Instead, public health initiatives focused on educating parents and adolescents on the timely management of testicular torsion should be explored as a mechanism to improve the testicular salvage rates.

It was also found that patients during the COVID-19 pandemic had longer mean ischemia times and lower testicular salvage rates compared with the preceding years. These findings are consistent with the results of a multicenter study in 2021 that assessed the time from symptom onset to ED presentation before and during the COVID-19 pandemic (15). Holzman et al. found that patients with testicular torsion during the pandemic waited longer to present to the ED after symptom onset and similarly reported delaying care. These findings suggest that patient concern for COVID-19 exposure could play a role in the timing of patient presentation and, consequently, the testicular salvage rates. As the time to hospital presentation is equally critical to the ischemia time, future QI initiatives and public health messaging that target reducing the time to presentation amidst a pandemic have the potential to increase the likelihood of testicular salvage.

The challenges in the implementation of the “code torsion” pathway were rooted in the interdisciplinary nature of the testicular torsion management, which required continuous education and resource investments from various departments. For example, it took over a year and half to establish hiring support in order to achieve full-time US technician coverage for weekends and overnights. Similarly, high turnover in the ED and on urology services contributed to the suboptimal awareness of the “code torsion” protocol. Another barrier to successful implementation was the provider knowledge of the protocol. Therefore, emphasis on departmental education surrounding “code torsion” was, and continues to be, integral to the success of this clinical pathway. An additional barrier considered by the multidisciplinary workgroup was the house staff call structure of home call to ensure that they were called into the hospital early on upon “code torsion” activation.

The current study has several limitations inherent to its use of retrospective data for QI. Data collection was dependent on accurate ICD-10 coding, which may not adequately capture all cases of testicular torsion. Moreover, granular clinical data on testicular salvage success at longer-term follow-up were lacking. The ischemia time was also determined using self-reported patient data documented in the electronic medical record, which may be affected by recall bias. Since clinical decisions to proceed with orchiectomy are surgeon-specific, the testicular salvage rates are subject to selection bias. The small sample size also limited the power of our study to detect statistically significant differences. Despite these limitations, this study uniquely contributes to the existing literature describing QI interventions for testicular torsion as we created a “code torsion” pathway based on scientifically established QI and change management methodology. Moreover, our protocol identified challenges, such as insufficient in-house radiology and OR staffing, which may or may not be unique to our institution. That said, our approach to studying the root causes of any delays in the door-to-incision time and implementing an iterative methodology to improve this metric might serve as a model for other institutions that might experience similar barriers to QI implementation.

In conclusion, we designed and studied a QI protocol for testicular torsion through iterative assessment and intervention. The “code torsion” pathways reduced the time from presentation to surgical intervention for testicular torsion. The testicular salvage rates were found to be associated with the total ischemia time and did not differ after QI implementation. While standardized clinical pathways for patients with suspected testicular torsion might improve the time to surgical intervention, further research is needed to understand their potential impact on the testicular salvage rates. Moreover, public health interventions targeting the pre-hospital timeframe are warranted to reduce the delays in presentation and the associated prolonged ischemia times.

## Data availability statement

The raw data supporting the conclusions of this article will be made available by the authors, without undue reservation.

## Ethics statement

Ethical approval was not required for the study involving humans in accordance with the local legislation and institutional requirements. Written informed consent to participate in this study was not required from the participants or the participants’ legal guardians/next of kin in accordance with the national legislation and the institutional requirements.

## Author contributions

SR: Data curation, Formal analysis, Investigation, Methodology, Resources, Software, Validation, Visualization, Writing – original draft, Writing – review & editing. KH: Data curation, Formal analysis, Investigation, Methodology, Supervision, Validation, Visualization, Writing – original draft, Writing – review & editing. TB: Conceptualization, Data curation, Formal analysis, Investigation, Methodology, Project administration, Resources, Supervision, Validation, Writing – review & editing. BF: Conceptualization, Data curation, Formal analysis, Investigation, Methodology, Project administration, Resources, Validation, Writing – review & editing. RS: Conceptualization, Data curation, Investigation, Methodology, Project administration, Writing – review & editing. SL: Conceptualization, Investigation, Methodology, Project administration, Supervision, Writing – review & editing. JS: Conceptualization, Formal analysis, Funding acquisition, Investigation, Methodology, Project administration, Supervision, Validation, Writing – review & editing.

## References

[1] Zhao LC Lautz TB Meeks JJ Maizels M. Pediatric testicular torsion epidemiology using a national database: incidence, risk of orchiectomy and possible measures toward improving the quality of care. J Urol. (2011) 186:2009–13. doi: 10.1016/j.juro.2011.07.024 21944120

[2] Sharp VJ Kieran K Arlen AM. Testicular torsion: diagnosis, evaluation, and management. Am Fam Physician. (2013) 88:835–40. Available at: https://www.aafp.org/pubs/afp/issues/2013/1215/p835.html. 24364548

[3] Gold DD Lorber A Levine H Duvdevani M Landau EH. Door to detorsion time determines testicular survival. Urology. (2019) 133:211–5. doi: 10.1016/j.urology.2019.08.003 31408640

[4] Zee RS Bayne CE Gomella PT Pohl HG Rushton HG Davis TD. Implementation of the accelerated care of torsion pathway: a quality improvement initiative for testicular torsion. J Pediatr Urol. (2019) 15:473–9. doi: 10.1016/j.jpurol.2019.07.011 31444122

[5] Heckscher D Jalfon M Buck MB Abello A Nguyen JV Casilla-Lennon M. Implementation of a health system intervention to reduce time from presentation to surgical intervention for pediatric testicular torsion. J Pediatr Urol. (2023) 20(2):254.E1–7. doi: 10.1016/j.jpurol.2023.10.042 38030428

[6] Powell T Sammut-Bonnici T. Pareto analysis. (2014) 12. doi: 10.1002/9781118785317.weom120202

[7] Taylor MJ McNicholas C Nicolay C Darzi A Bell D Reed JE. Systematic review of the application of the plan–do–study–act method to improve quality in healthcare. BMJ Qual Saf. (2013) 23:290–8. doi: 10.1136/bmjqs-2013-001862 PMC3963536 24025320

[8] Barbosa JA Tiseo BC Barayan GA Rosman BM Miranda Torricelli FC Passerotti CC. Development and initial validation of a scoring system to diagnose testicular torsion in children. J Urol. (2013) 189:1859–64. doi: 10.1016/j.juro.2012.10.056 23103800

[9] Arevalo MK Sheth KR Menon VS Ostrov L Hennes H Singla N. Straight to the operating room: an emergent surgery track for acute testicular torsion transfers. J Pediatr. (2018) 192:178–83. doi: 10.1016/j.jpeds.2017.09.009 PMC5737783 29246339

[10] Afsarlar CE Ryan SL Donel E Baccam TH Jones B Chandwani B. Standardized process to improve patient flow from the Emergency Room to the Operating Room for pediatric patients with testicular torsion. J Pediatr Urol. (2016) 12:233. e1–233. e4. doi: 10.1016/j.jpurol.2016.04.019 27270069

[11] Madsen SMD Rawashdeh YF. Assessing timeline delays associated with utilization of ultrasound diagnostics in paediatric acute scrotum, pre and per COVID-19 pandemic. J Pediatr Urol. (2023) 19:653 e1–7. doi: 10.1016/j.jpurol.2023.07.003 37544787

[12] Cost NG Bush NC Barber TD Huang R Baker LA. Pediatric testicular torsion: demographics of national orchiopexy versus orchiectomy rates. J Urol. (2011) 185:2459–63. doi: 10.1016/j.juro.2011.01.016 21527194

[13] Bayne AP Madden-Fuentes RJ Jones EA Cisek LJ Gonzales Jr ET Reavis KM. Factors associated with delayed treatment of acute testicular torsion—do demographics or interhospital transfer matter? J Urol. (2010) 184:1743–7. doi: 10.1016/j.juro.2010.03.073 20728168

[14] Bayne CE Villanueva J Davis TD Pohl HG Rushton HG. Factors associated with delayed presentation and misdiagnosis of testicular torsion: a case-control study. J Pediatr. (2017) 186:200–4. doi: 10.1016/j.jpeds.2017.03.037 28427778

[15] Holzman SA Ahn JJ Baker Z Chuang K-W Copp HL Davidson J. A multicenter study of acute testicular torsion in the time of COVID-19. J Pediatr Urol. (2021) 17:478 e1–6. doi: 10.1016/j.jpurol.2021.03.013 PMC7977032 33832873

